# Exploring drivers for safe male circumcision: Experiences with health education and understanding of partial HIV protection among newly circumcised men in Wakiso, Uganda

**DOI:** 10.1371/journal.pone.0175228

**Published:** 2017-03-31

**Authors:** Simon P. S. Kibira, Marguerite Daniel, Lynn Muhimbuura Atuyambe, Fredrick Edward Makumbi, Ingvild Fossgard Sandøy

**Affiliations:** 1 Centre for International Health, Department of Global Public Health and Primary Care, University of Bergen, Bergen, Norway; 2 Department of Community Health and Behavioural Sciences, Makerere University School of Public Health, Kampala, Uganda; 3 Department of Health Promotion and Development, University of Bergen, Bergen, Norway; 4 Department of Epidemiology and Biostatistics, Makerere University School of Public Health, Kampala, Uganda; Cardiff University, UNITED KINGDOM

## Abstract

**Introduction:**

About 2.5 million men have voluntarily been circumcised since Uganda started implementing the WHO recommendation to scale up safe male circumcision to reduce HIV transmission. This study sought to understand what influences men's circumcision decisions, their experiences with health education at health facilities and their knowledge of partial HIV risk reduction in Wakiso district.

**Methods:**

Data were collected in May and June 2015 at five public health facilities in Wakiso District. Twenty-five in-depth interviews were held with adult safe male circumcision clients. Data were analysed using thematic network analysis.

**Findings:**

Safe male circumcision decisions were mainly influenced by sexual partners, a perceived need to reduce the risk of HIV/STIs, community pressure and other benefits like hygiene. Sexual partners directly requested men to circumcise or indirectly influenced them in varied ways. Health education at facilities mainly focused on the surgical procedure, circumcision benefits especially HIV risk reduction, wound care and time to resumption of sex, with less focus on post-circumcision sexual behaviour. Five men reported no health education. All men reported that circumcision only reduces and does not eliminate HIV risk, and could mention ways it protects, although some extended the benefit to direct protection for women and prevention of other STIs. Five men thought social marketing messages were ‘misleading’ and feared risk compensation within the community.

**Conclusions:**

Participants reported positive community perception about safe male circumcision campaigns, influencing men to seek services and enabling female partners to impact this decision-making process. However, there seemed to be gaps in safe male circumcision health education, although all participants correctly understood that circumcision offers only partial protection from HIV. Standard health education procedures, if followed at health facilities offering safe male circumcision, would ensure all clients are well informed, especially about post-circumcision sexual behaviour that is key to prevention of risk compensation.

## Introduction

Safe male circumcision (SMC) is a one off long-term efficacious intervention for both individual and population HIV prevention [1–4] recommended in countries where its prevalence is low and the HIV epidemic is generalised [5, 6]. Studies published after the 2007 World Health Organization (WHO) recommendation to scale up SMC, have shown further benefits of SMC beyond HIV prevention; a reduction in the prevalence and incidence of high risk Human Papilloma virus [7, 8], incidence of Herpes Simplex [9], and genital ulcers in female partners of circumcised HIV negative men [10]. It also reduces the risk of penile cancer, prevents inflammation of the glans and the foreskin, makes it easier to keep the penis clean, and prevents potential development of scar tissue on the foreskin [11].

International agencies globally make use of targets to achieve health and development goals that motivate country governments to accelerate progress. Target five of the UNAIDS 2016–2021 strategy aims to have 27 million additional medical circumcisions conducted by 2020 in high HIV prevalence settings like Uganda, as part of integrated sexual and reproductive health services for men [12]. This is in line with sustainable development goal 3.3 that aims in part, to end the AIDS epidemic [13]. The Copenhagen consensus identified scaling up SMC to 90% of HIV negative men by 2030 as one of the best investments to realise the post-2015 development agenda. The estimated benefit is 28 US$ for every dollar invested [14] in HIV hyper endemic countries (with adult prevalence >15%). The benefit of investing in SMC is also high for countries with epidemic HIV [15, 16] such as Uganda.

Circumcision prevalence among men 15 to 49 years in Uganda was 27% in 2011 [17], but with high levels of willingness to be circumcised among uncircumcised men [18]. Those who expressed willingness at the time also seemed to be the ones with the largest need for protective measures [18]. SMC has since been promoted using mass media, posters, billboards, and automobiles with loudspeakers that drive through communities, especially when outreach services are planned. These methods help to mobilise men to come for the services. The public health facilities offering SMC have displayed logos indicating the availability of this free service while some private facilities also provide the services at a cost. In 2014, over 3.2 million males were circumcised in the 14 WHO-priority countries, bringing the cumulative total to 9.1 million men since the recommendation was first made, and Uganda was one of the better performers with 878,109 men circumcised. The circumcision prevalence among adult men in Uganda has been reported to be as high as 40% in 2014, with 2,114,461 men circumcised under the SMC program between 2010 and 2014 [19]. Only Kenya, Ethiopia and Tanzania achieved higher coverage [20].

WHO/UNAIDS recommends that provision of circumcision at health facilities should include health education and counselling for all men. There should be group education sessions for clients to have basic information on sexual and reproductive health, including HIV, before additional individual counselling is conducted. Circumcision-specific education should include information about the health benefits of SMC, how the procedure is conducted and potential complications as well as when to resume sex; it should also inquire about and correct myths among clients [11].

It is vital for SMC clients to appreciate that circumcision provides only partial protection against HIV. Such an understanding may motivate them to take other precautions beyond circumcision, such as using condoms and being faithful to one partner, to further reduce their chances of HIV infection. Since comprehensive information about partial HIV risk reduction is ideally provided as part of the health education [11] at health facilities, it is important to explore if and what messages, men remember after receiving SMC services. Men’s post circumcision behaviours may also be affected by the factors that influenced them to seek SMC. Previous studies have shown that among the most important reasons for deciding to circumcise are the expected benefits in terms of HIV risk reduction, and the influence of women[21–23]. However, these studies have been largely based on community views and male informants who have not yet been circumcised [21, 22, 24–27, 23]. Only a few included self-reports of circumcised men [28–30]. This study explored the drivers of adult men’s circumcision decisions from the perspective of SMC clients, their experiences with health education at health facilities and personal understanding of partial HIV protection that SMC offers.

## Methods

### Study location and participants

This qualitative study was conducted in Wakiso district, central Uganda. Wakiso district has a cosmopolitan population due to its proximity to the country’s capital city. It is the largest district in Uganda with 5.8% (1,997,418) of the national population recorded in the 2014 census [31]. Men comprised 48.2% (962,121) while the urban population was 59.2% (1,182,901) [31]. The district has 103 health facilities including four hospitals, five Health Centres (HC) IV, 37 HC III and 57 HC II offering varied services summarised in Table 1 [32]. SMC services are provided free of charge at public health facilities with operational theatres such as HC IV level, and through mobile outreach clinics in areas without surgical theatres.

**Table 1:** Summary of services offered at different facility levels

Facility level	Coverage	Services offered
HCII	Parish level	Preventative, promotive & outpatient curative services
HCIII	Sub-County	HCII services plus maternity, in-patient care, laboratory services
HCIV	County level	HCIII services plus blood transfusion and emergency surgery services
General/district hospital	District level	HCIV services plus in-service training, consultation and research to community based healthcare programmes

We purposively selected adult men who came for SMC at public health facilities offering this service in the district from May to June 2015. The men were selected from five level III and IV health facilities. The eligibility criteria for the men included being an adult aged 18 to 59 years, able to give written informed consent, married or having a stable partner at the time of the initial interview, and seeking SMC voluntarily. Eligible participants were recruited at the health facilities through health workers who informed them when they came for SMC about the possibility to participate in the study. After indicating a willingness to participate, they were approached by the researchers who explained further study details and provided them with a written informed consent form.

Twenty-five men were recruited and all were married or had stable sexual partners at the time of the interview. Six men reported two or more partners/wives. Fifteen men were aged above 24 years and participants’ ages ranged from 18 to 46 years. Due to the cosmopolitan nature of the district, participants were from seven ethnic backgrounds found in Central (Baganda, Baluri), Eastern (Basoga, Bateso) and Western (Banyankore, Bakiga, Banyarwanda) parts of Uganda. Fourteen lived in rural areas and twelve men were circumcised at HC IV level centres while the rest were circumcised at HCIII facilities and outreach points. Twelve men had primary education, nine had secondary, while four had tertiary/university education as the highest level attended.

### Data collection and analysis

We conducted 25 in-depth interviews with men in Luganda (district main language), Runyankore/Rukiga (spoken in south western Uganda) and Lusoga (spoken in parts of eastern Uganda) languages. All interviews were held either on the same day after receiving the SMC service at the health facility premises, or one day later at their respective homes if the informant preferred. An interview guide was designed to discuss informants’ motives for SMC, what influenced uptake, experiences with health education received (if any), and understanding of partial risk reduction among other issues. In-depth interviews were used owing to the sensitivity of the research questions and the need to explore personal experiences.

All interviews were recorded using digital voice recorders, transcribed and translated into English. Complete transcripts were imported into atlas.ti7.5 qualitative data management software (Scientific Software Development GmbH) for analysis. Data were analysed using a thematic approach. We utilised thematic networks [33] to systematise the extraction of basic themes, organising themes and global themes. The basic themes are the lowest order premises that are derived from the data while organising themes are more abstract middle-order themes that categorise basic themes into clusters of similar issues. Global themes in the case of this study are the overarching themes that show the components of the study aim [33].

The coding procedure involved three people who generated a coding framework that was applied to the rest of the transcripts while allowing for new codes to emerge. Codes were discussed until consensus was reached. Basic themes were then identified from the coded segments of data and refined. The basic themes were then rearranged into organising themes, and three global themes that reflected the research questions for this paper were deduced. Each global theme reflected similar organising themes about a specific issue. The thematic networks are presented describing contents under each basic theme with support from text segments of data where necessary. (Table 2).

**Table 2:** An example of the basic, organising and global themes emerging from the coding.

Global themes	Organising themes	Basic Themes
Drivers for SMC decisions	Personal need to reduce HIV/STI risk	• Reduce the chances of HIV infection. • Reduce risk of STIs.
	Influence of sexual partners in circumcision decision	• Partner directly wanted it. • Partner talked about this often indirectly • Perceived enhanced sexual performance. • Women prefer circumcised men. • Mistrusting partner’s sexual behaviour. • Protect the partner from infection risk. • Perceived better penis appearance.
	Personal hygiene	• Easy to clean sexual organs • Tired of dirt under foreskin
	Positive community perceptions	• Fashionable/trendy • Influence of friends and family. • Influence by other circumcised men
	Timing of circumcision	• Waited until my partner was away. • Waited until I had leave • Waited until a period with less work.
Experience with health education at the health facilities	Health education about surgical procedure and healing.	• Wound healing. • Waiting period. • SMC procedure. • Demonstrated how the procedure is done.
	HIV/STI risk reduction and other benefits of SMC.	• SMC reduces HIV risk • SMC reduces STIs risk. • Reduces cancer risk to women. • Other benefits
	Post healing sexual behaviour and HIV Testing	• Sexual behaviour after healing • Received free condoms. • HIV testing before SMC.
	Poor quality or absent health education	• Too anxious to listen to all messages. • The time was not enough. • They told us nothing
Personal understanding of partial protection for HIV after SMC	Knowledge on how HIV risk is only reduced but not eliminated	• It only reduces HIV risk. • It reduces the risk of STIs. • Reduce HIV re-infection (other strains). • Removes foreskin that harbours HIV. • Limits bruising in intercourse. • It protects the man more than a woman.
	Misunderstanding HIV risk reduction	• Reduces HIV risk from man to woman. • Absolute protection from “minor” STIs.
	Unconvinced about risk reduction messages	• Fear that some men will compensate for perceived reduced risk. • The focus should be put on other benefits. • Not convinced of HIV risk reduction. • SMC messages not well understood.

### Ethical considerations

The study protocol was assessed in accordance with the Norwegian Research Ethics Act and the Health Research Act. It was thereafter exempted from review by the Regional Ethical Committee of Western Norway (reference 2015/477) in March 2015 because it did not involve experiments on human subjects. It was reviewed and approved by the Higher Degrees, Research and Ethics Committee at Makerere University School of Public Health (registration 288) in April 2015. The study was then approved and registered by the Uganda National Council for Science and Technology (SS 3764) in May 2015. Permission to collect data was obtained from the Wakiso District health office and the health facilities where men were recruited. Further, the purpose of the study, the extent of their involvement and rights were explained to all study participants before obtaining their written informed consent. Consent included to anonymously use their interview data in the study reports. All audio recordings of the interviews were erased after transcription was completed. Study participants were compensated 20,000 Uganda shillings (about US$ 7) for their time.

## Findings

Findings are presented using three global themes (Table 2): (1) Drivers for men’s circumcision decisions; (2) experiences with health education at the health facilities; and (3) men’s understanding of partial HIV risk reduction. Under each global theme, the organising and basic themes are presented with typical and deviant quotations used to illustrate some of the participants’ accounts.

### Drivers for men’s circumcision decisions

This global theme comprises five organising themes: personal need to reduce the risk of HIV/STIs, the influence of sexual partners, personal hygiene, positive community perception of male circumcision and timing of circumcision.

A strong desire to reduce the personal risk of infection with HIV and STIs such as gonorrhoea and syphilis was an important factor in decisions to seek SMC. Nearly all the men reported that they knew that circumcision reduced their risk and recognised a fear for such infections.

*I fear contracting HIV and they say circumcision reduces the chances of infection*. *So why don’t I circumcise with this big benefit*? *I also talked to my “women”* [he has two partners] *and we agreed with them*. *(P17*, *age 41*, *secondary education)*.

The men had received information about risk reduction for HIV/STIs from community mobilisation using community radio in the trading centres nearby and motor vehicles from the health facilities with loudspeakers and SMC mobilisation messages. Many men had also heard and read messages from the print and electronic media such as radio, television, and newspapers. Only those with secondary and tertiary education reported reading newspapers. They had also received information from friends in the communities, circumcised colleagues, as well as directly from health workers.

I heard on the radio several times that circumcision helps to prevent some of the STIs. I used to have recurrent blisters on the penis and I decided to come. That was my biggest challenge. I thought maybe this problem will go. I also hear that it prevents gonorrhoea. (P9, age 30, primary education).

Men’s motivation to seek circumcision was strongly related to their sexual partners. Women influenced decisions even when the men had prior knowledge regarding risk reduction for HIV. Most men reported that their partners directly expressed a desire to see them circumcised, while some men mentioned that their partner had expressed her wishes by referring to circumcised men as clean, less prone to infections, less likely to infect women with cervical cancer, and having better penis appearance (in one case). Men believed that women obtained this information from within the community or from health workers while receiving other services like antenatal care.

*We had talked about it for some time*. *She used to ask me “why don’t you go for circumcision*?*” and I would ask her back*, *why do you always ask me to go for it*? *Then she told me “you go for it and you will see my reasons*.*” Now I have just called to tell her* [lives upcountry] *and she is very excited about it*. *(P1*, *age 25*, *secondary education)*.

*Nalongo* [one of his wives] *one time told me that if I ever knew of cervical cancer screening programmes going on at the health facility*, *I should let her know*. *She said they told them at the facility that an uncircumcised man has high chances of infecting their partner with a cervical*, *cancer-causing virus*. *She jokingly asked me “when will you ever be courageous to go for circumcision since you were able to take your sons*?*” That is why I am here*. *I think they all* [his other wives] *wanted it but they would not come out directly like she did*. *(P10*, *age 46*, *primary education)*.

Half of the men, irrespective of age, perceived circumcision to enhance sexual performance and expected to better satisfy their partners. They reported that it was common knowledge that women in the community and elsewhere preferred circumcised sexual partners. This widespread perception played a role in many decisions to seek circumcision to increase sexual appeal.

*Sometime back*, *I read from New Vision or Bukedde* [local daily newspapers] *about sexual performance*. *That the glans is very sensitive and when the foreskin retracts*, *it makes it much too sensitive for a man to last long during intercourse*. *Of course*, *any man would like to enhance sexual performance*. *That if you get circumcised*, *that skin will be hardened and it will be less sensitive during intercourse*. *(P8*, *age 40*, *tertiary education)*.

I heard from my friend. He told me that sexual pleasure will be different once you are circumcised. That is what I know. He said that ‘your partner will be more satisfied and even if she cheated on you with an uncircumcised man, she will still come back to you because you are certainly better.’ (P13, age 21, primary education).

Men who had informed their partners about the decision to circumcise in advance said their partners were excited about it, giving them the courage to turn up. Interestingly, some men did not inform their partners beforehand because they wanted to surprise them on their return. These men reported that their partners doubted they would adhere to their wishes or doubted their courage to bear the perceived surgical pain.

*It has taken about three months now* [to comply with her wishes]. *But today I decided to give her a surprise because she is visiting her parents in the village*. *I want her to come back when “everything is new”*. *She is not expecting this at all because I have not done it for all the months we discussed it*. *It will be a complete surprise*. *I don’t want to tell her yet*. *I want her to find when things are well as she has always wanted*. *(P6*, *age 24*, *primary education)*.

Three men sought circumcision to protect their sexual partners from the risk of infection with cervical cancer and HIV; this was in addition to other reasons already reported above.

*For me*, *the very first reason was to reduce the chances of getting infected with HIV*. *The second reason was to reduce the dirt that builds up under the foreskin and also to reduce the chances of my partner getting infected with those cancers I have told you* [cervical cancer]. *(P1*, *age 25*, *secondary education)*.

Four men mistrusted their partners’ sexual behaviour and partly sought circumcision to have more protection in case of any infection from partners’ extra marital sex.

Another driver was personal hygiene. Many men reported that this was a reason mentioned by their partners or wives in order to persuade them, and they also cited this reason as something that contributed to their decision to seek circumcision.

I have been personally challenged about this even before my wife influenced me. If you do not bathe in the morning and you wait until the evening, you will be surprised how dirty underneath your foreskin will be. In today’s slang language one would say “you also fear yourself.” You have to shower twice a day, yet circumcised men can shower once a day if they want without much trouble. I was tired of this. (P22, age 41, secondary education).

There was a strong wave of community positivity about circumcised men in general, which influenced men’s desire for circumcision. In relation to this, circumcised friends of the participants within the wider community also used their experience to influence them, allaying fears about perceived surgical pain. In men’s social gathering places like bars and pool halls, and places of work like construction sites, circumcision was reportedly discussed. One quarter of men also noted that many people they knew thought that circumcision was fashionable.

I will give you several reasons why we come. But I think mainly because it is fashionable to be circumcised nowadays. It is the trend that many women want men to take. I would say it is like a woman telling you ‘please wear shorter socks, they are trendy, or you should not wear hemmed trousers.’ It is the way to go now. (P8, age 40, tertiary education).

After taking the decision to circumcise, the timing of when to have the surgery was an important factor. It was crucial for seven men to go for SMC at a time when either they or their sexual partners were away from home for some days or weeks. This was to ensure healing free from “sexual temptations.” The presence of their partners was perceived as an undesirable disturbance that would cause unwanted erections with sutured sexual organs and delay healing. Three men also ensured that they waited until they had a period of limited or no work. They were casual labourers in the construction industry who had postponed their decision, fearing to lose productive time.

I decided to wait until she went to the village; away from me because now it will be easier for me to heal without any temptations. I will not be thinking so much about her when she is away. I have heard from circumcised men that when you think about a woman during the healing period, you get a lot of pain. This will delay your healing. So I had to take this chance too. (P6, age 24, primary education).

### Experiences with health education at the health facilities

We explored the experiences of men when they sought SMC services at health facilities regarding the kind of messages that they received. Results here are presented using four organising themes: Health education about surgical procedure and healing; HIV/STI risk reduction and other benefits of SMC; post-healing sexual behaviour and HIV Testing; poor quality or absent health education.

Twenty men reported that they received some kind of health education comprising how the circumcision surgical procedure was going to be done, the length of the healing period, care for the wound and a clear warning not to resume sex before the communicated healing period was over. The procedure messages included allaying fears of the perceived pain that men expected to have, reassurance about the use of anaesthesia and in some instances showing pictures of the different forms of circumcision procedures that can be performed. Men were also cautioned to follow the guidelines for proper healing in varied ways. While some were told to only take oral pain killers provided, keep the wound dry and not to apply any other substances, others were told to use “lukewarm salty water to carefully clean the wound.” Only a few were told to return to the facilities for review. They were also cautioned to do less manual work and avoid long travel in the first few days.

They told us that we are not supposed to have a full body shower for a whole week; we can only bathe partially, carefully wiping the body and the groin area near the wound. Then after a week, if you want to shower, you get a clean clear polythene bag, wrap the private parts and bathe. (P3, age 30, primary education).

The six weeks waiting period had been emphasised and caution was given not to have sex even when they visibly appeared healed before the prescribed period. In a few instances, men shared that they were also provided with phone contacts of health workers in case they had any challenges during this phase.

The main message was caution not to resume sexual intercourse before the healing period of six weeks. I think no man will complain when they have problems because the health workers were very clear on this message to all of us. Other clients were young boys. But anyone who had a girlfriend was cautioned on this. The other message was about keeping proper hygiene throughout the healing period and after. (P19, age 33, primary education).

Five men among the 20 who received pre-SMC health education, said they remembered only a few messages due to anxiety because *“as they explain to you*, *your mind is focused on the possible pain from the surgical blade and how the procedure will go*.*” (P10*, *age 46*, *primary education)*. Only nine said they were cautioned on the importance of safe sexual behaviour after healing. They were encouraged to use condoms and/or avoid multiple sexual partnerships because circumcision does not offer full protection. Ten were offered an HIV test prior to being circumcised, and one received condoms.

They told us about how to behave after circumcision. They told us that circumcision alone will not prevent HIV completely or other STIs, but it reduces the risk by 60%. So we were encouraged to behave well. They told us about how to behave during the healing so that we have no complications. (P20, age 28, tertiary education).

Health education also emphasised the role of circumcision in HIV/STI risk reduction. Men were told that SMC reduced their risk of HIV infection and other STIs with some health workers reportedly emphasising the 60% risk reduction while others did not mention percentages but emphasised that it was only partial. Other benefits of SMC discussed included a reduction in cervical cancer risk to women and hygiene related benefits.

He told me about all the advantages, the diseases that it prevents, like penile cancer and cancer of the cervix for women, syphilis. He also talked about HIV. (P8, age 40, tertiary education).

It is worth noting that five men said they did not receive any kind of health education at the health facilities prior to circumcision, although some of them received HIV testing services. One man said the session was rushed and that he had no opportunity to ask questions. This excerpt gives an example of a young man who received no health education:

They did not tell us anything…

*We came here* [with two colleagues] *and told them we have come for circumcision*. *They told us to buy books* [for records], *and then showed us the health worker responsible*, *who wrote something in the books and sent us to the lab for HIV testing*. *We were tested and received results*. *We were then told to come back to the theatre and that was it*. *We were circumcised*.

I: Why do you think they did not explain anything about what was happening?

P: Maybe the counsellors are not around. I do not know. You know what happens with our public health centres. Anyway, we wanted circumcision and got it. (P11, age 21, primary education).

### Personal understanding of partial risk reduction for HIV after SMC

We also explored how men understood the concept of partial risk reduction for HIV after receiving SMC services. This was important because such knowledge may influence sexual behaviour post circumcision. Men’s accounts were organised into: those that understood how HIV risk is only reduced but not eliminated, misunderstanding of HIV risk reduction, and being unconvinced about risk reduction messages.

Twenty-two of the men were able to explain that circumcision only reduced the risk of HIV infection. They acknowledged the possibility that one could be infected and thus needed to continue taking precautions after SMC. Some specifically mentioned the 60% risk reduction conveyed in the ideal health education messaging at facilities but explained it in their own terms.

*I heard it reduces* [HIV risk] *by 60%*. *This means that the 40% chances* [to be infected] *are still there*. *Even in a football game*, *if one team has 70% ball possession*, *they may still lose the game when the other team has only 30% of the ball*. *That is how I relate this risk reduction in normal life*. *It means you can still get infected in case you do not use condoms if you think you will depend on the 60% chances alone*. *It means you still have to protect yourself when you know you are HIV negative*. *We all want life*. *Especially when you are still a young person and have no child in life yet*. *(P11*, *age 21*, *primary education)*.

Most explained that the removal of the foreskin, which could harbour the virus after sexual intercourse, was crucial in reducing the chances of getting HIV infection. The foreskin, they said, provides a conducive, warm environment underneath for the viruses to thrive before entering the body. Such information was received from health education and from other sources in the community and did not differ by educational level or age of the men. Related to the foreskin, half of the men said the removal of the skin exposes the glans and hardens it, limiting chances of bruising during less lubricated intercourse.

Because this foreskin is now removed, the head of your penis becomes hardened. This means you have reduced your chances of the virus entering your skin. When your penis head is hardened, you cannot have bruises on it; you get rid of all these potential damages to the skin that you would get if you were uncircumcised. It is so easy to get bruises on the skin when you engage in sexual intercourse with your partner if uncircumcised. But this is very hard for a circumcised man because you cannot bleed. When you are uncircumcised you bleed because you get bruised and this is a big risk for HIV infection. (P1, age 25, secondary education).

However, there were some important misconceptions from four men. One man reported that he knew circumcision also directly reduced HIV risk to a woman, while another two reported that it provides absolute protection from what they called minor STIs. Both men had secondary education. One man also said that after circumcision, he could just wipe or wash the penis when he had condom-less sex as a measure to further reduce infection risk.

I have hope that finally my dream of preventing STIs that I feared, most especially gonorrhoea, is now realized. I am taking this [circumcision] as being vaccinated against those STIs, as I always wanted. (P5, age 29, secondary education).

Five men (all with secondary or tertiary education) were unconvinced that circumcision reduces HIV infection risk. Even though they were aware of the messages, in their own view, these were “misleading” and not well understood by some people. They recommended that mobilisation messages focus on other SMC benefits like improved hygiene, with HIV risk reduction as an additional benefit. They also feared that some men indeed do or will *“approach the football field without shoes”* [have condom-less sex], believing they are fully protected. Similarly, four men also felt that they were unable to explain how the HIV infection risk is reduced, although they said knew it was not full protection.

I do not believe that circumcision will reduce the chances of the virus entering through the urethra because it still remains as open as that of an uncircumcised man. It is very hard to convince me that the 60% works. Even if the glans is hardened, it is not too hard for nothing to enter. But today I did not challenge the doctor because you know you cannot challenge health workers. But no one can confirm that 60%. How do you confirm that the percentage is 60%? So I did not listen to this 60%. I am not convinced. (P8, age 40, tertiary education).

## Discussion

This study explored the main influences of adult men’s circumcision decisions, experiences with health education at health facilities and their knowledge of HIV risk reduction from circumcision. Female sexual partners played a leading role in influencing respondents’ decision to seek SMC, although a reduction of HIV risk was also important. During health education, more emphasis was perceived to be put on wound care and the surgical procedure, as well as benefits of SMC while a few of the men reported a focus on post SMC sexual behaviour. All the men, however, were aware that circumcision only offers partial risk reduction for HIV infection. Nonetheless, there were a few who in addition to this, wrongly thought male circumcision reduces the transmission risk from man to woman and/or entirely eliminates STI transmission risk.

Men reported both direct and indirect ways that their partners influenced them to seek SMC. The direct influence was where the partners explicitly told their husbands or men that they preferred them circumcised. Indirect influence included cases where the partners discussed circumcision to be beneficial in varied ways without directly telling the men to go for it. In patriarchal societies like Uganda, matters concerning men’s sexual health may be one of the few areas where women have such strong influence. A study in Zambia found that women’s acceptance of circumcision and discussion with partners influenced men’s readiness to undergo SMC [25, 22] and in Kenya, a study documented that some women who were ‘more knowledgeable’ about circumcision educated their partners and encouraged them to go for the service [34]. Studies in Botswana and Tanzania showed both direct and indirect influence as well, with women using “soft” language to convince partners, mindful not to endanger their marriages or relationships, while others even denied partners sex to effect circumcision decisions [28, 29]. However, in contrast to this, in a study in Rakai, Uganda [35], conducted before the national scale up of SMC, female partners were reported as deterring rather than motivating the decision to get circumcised.

Men in this study believed that circumcision enhanced sexual performance and perceived women to prefer circumcised men when making sexual partner choices. Such influence through beliefs about sexual performance has also been reported in many studies in WHO priority SMC countries [30, 28, 21, 36, 29, 37–39] and elsewhere [40]. Perception of partner preference for circumcised men has also been reported in other places [34, 24, 41, 26, 40, 42]. These widespread perceptions are also most likely influenced by the SMC social marketing campaigns. For example, messages from the “Stand Proud, Get Circumcised” campaign in Uganda [43] aimed to use women as key players in influencing men, and portrayed SMC as one of the attributes of a “modern stylish man” [44]. Preference for circumcised men is also listed as an additional benefit in some brochures stating “women believe a circumcised penis looks better” and “possibly gives greater sexual satisfaction” [45]. This could explain the generally positive community perception towards circumcision reported in this study.

HIV infection risk reduction was the second most mentioned reason to seek SMC, after their partners’ influence. It is not surprising because this relates to the central message of SMC social marketing. All men had heard such messages in the media, within the community and/or at health facilities. Other reasons reported such as better penile hygiene, risk reduction to some STIs and cervical cancer prevention to partners, are also portrayed in SMC messages to the public in Uganda [45, 46]. Studies conducted in other sub Saharan countries [47, 48, 27, 29, 26] have also reported that these reasons play a role in influencing men to circumcise.

Although rare, it is worth noting that some men reported that they received no health education from health facilities. Even among those that experienced health education, some said they were not told about post SMC desired sexual behaviours. These reports indicate gaps in how the WHO recommendations regarding pre SMC health education are followed [11]. This could be due to competing services provision in hospitals and HCs with large numbers of patients where health workers have multiple demands and limited time. Men who were circumcised at facilities with health workers dedicated to providing SMC reported more detailed health education and the offer of HIV testing. Given the fears of behavioural risk compensation and the potential dangers this may pose for HIV infection [49], such gaps in health education are a point of concern. Furthermore, the appropriate time to provide health education is also important. Some men experienced anxiety prior to surgery that affected how much they grasped from the health education information they were given. Providing extra sexual behaviour related messages during the post-operative period could probably be helpful.

All participants understood that SMC did not yield 100% protection from HIV infection and identified the need for maintenance or adoption of safer sexual behaviours after the procedure, such as condom use. Such knowledge was also reported after a four-year scale up in Rakai, Uganda [50], and in Kisumu, Kenya [51], although in these clinical trial areas, the health education was probably more comprehensive than in programme settings. Clinical trial settings are study environments that are well organised, with strict adherence to protocols. This may not be the case in programme settings where other competing interests may affect adherence to guidelines. In this study, we expected that men would obtain health education information from staff at the respective health facilities. However, many had instead received health information from other sources such as radio and television social marketing, from peers, as well as circumcised friends. Participants who reported no “formal” health education from health facilities also managed to obtain such information.

A few men reported misconceptions as well, thinking SMC also reduced the risk of HIV transmission from men to women directly and, absolutely protected them from other STIs. Concerns about misunderstanding the level of protection from circumcision have also been reported in southern Africa [52, 53] and among fishing communities in Uganda [27]. Such misconceptions could be due to information from less reliable sources within communities and/or misunderstandings of SMC social marketing messages, as indeed feared by some men in this study.

The strength of this study is that the findings coincide with research conducted on SMC programmes in other countries. There are several limitations however, that should be considered when interpreting the findings in this study. The study was conducted in only one district in central Uganda, although participants came from various cultural backgrounds from other parts of Uganda. Conceptual generalisation may, therefore, be limited. Although men were interviewed on the day of receiving services or one day later, there is a possibility of an inaccurate recall of what transpired during the health education sessions. Some participants acknowledged pre surgical anxiety affecting what they could grasp. Although we explained that there was no direct link between the research team and the health system, some participants occasionally referred to interviewers as “musawo” (meaning health worker). It is, therefore, possible that some could have reported what they thought the ‘health worker’ would like to hear.

The participants reported positive community perceptions about SMC campaigns, influencing them to seek services and enabling female partners to impact this decision-making process directly or indirectly. Partner involvement can be enhanced to go beyond influencing decision making for SMC. It can include physical presence of the partners at the health facilities for joint health education where agreeable and help in the maintenance of safer sexual behaviour post circumcision. The SMC programme could also provide couples reproductive health services such as couple HIV counselling and testing at SMC points.

There appeared to be gaps in SMC health education at some health facilities based on what the men reported, with the main focus placed on the immediate concerns of the surgery and the healing process, and less focus on the post SMC safer sexual behaviour. However, it is encouraging that all participants correctly understood that SMC offers only partial protection from HIV even though a few stretched the direct protection to women and to STIs.

Standard health education procedures, including counselling post-surgery, if followed at all health facilities offering SMC, would help in informing all the clients, especially about post SMC sexual behaviour that is key to prevention of risk compensation or misinformed risky behaviour. This is important because there are many other, probably less reliable, sources of information in the communities that could mislead some men.

## Supporting information

S1 Text Excerpts from the interviews. (DOCX)

## References

[1] Gray RH, Kigozi G, Serwadda D, Makumbi F, Watya S, Nalugoda F et al Male circumcision for HIV prevention in men in Rakai, Uganda: a randomised trial. Lancet. 2007;369(9562):657–66. 10.1016/S0140-6736(07)60313-4 17321311

[2] Bailey RC, Moses S, Parker CB, Agot K, Maclean I, Krieger JN et al Male circumcision for HIV prevention in young men in Kisumu, Kenya: a randomized controlled trial. Lancet. 2007;369(9562):643–56. 10.1016/S0140-6736(07)60312-2 17321310

[3] Auvert B, Taljaard D, Lagarde E, Sobngwi-Tambekou J, Sitta R, Puren A. Randomized, controlled intervention trial of male circumcision for reduction of HIV infection risk: the ANRS 1265 Trial. PLoS medicine. 2005;2(11):e298 10.1371/journal.pmed.0020298 16231970 PMC1262556

[4] Mehta SD, Moses S, Agot K, Odoyo-June E, Li H, Maclean I et al The long-term efficacy of medical male circumcision against HIV acquisition. AIDS (London, England). 2013;27(18):2899–907. 10.1097/01.aids.0000432444.30308.2d 23835501

[5] WHO/UNAIDS. New Data on Male Circumcision and HIV Prevention: Policy and Programme Implications. Geneva, Switzerland: World Health Organisation and Joint United Nations Programme on HIV/AIDS,2007 March 28.

[6] WHO/UNAIDS. Joint Strategic Action Framework to Accelerate the Scale-Up of Voluntary Medical Male Circumcision for HIV Prevention in Eastern and Southern Africa 2012–2016: World Health Organisation and Joint United Nations Programme on HIV/AIDS,2011 November.

[7] Grabowski MK, Kong X, Gray RH, Serwadda D, Kigozi G, Gravitt PE et al Partner Human Papillomavirus Viral Load and Incident Human Papillomavirus Detection in Heterosexual Couples. The Journal of infectious diseases. 2016;213(6):948–56. 10.1093/infdis/jiv541 26597261 PMC4760424

[8] Auvert B, Sobngwi-Tambekou J, Cutler E, Nieuwoudt M, Lissouba P, Puren A et al Effect of male circumcision on the prevalence of high-risk human papillomavirus in young men: results of a randomized controlled trial conducted in Orange Farm, South Africa. The Journal of infectious diseases. 2009;199(1):14–9. 10.1086/595566 19086814 PMC2821597

[9] Tobian AA, Serwadda D, Quinn TC, Kigozi G, Gravitt PE, Laeyendecker O et al Male circumcision for the prevention of HSV-2 and HPV infections and syphilis. The New England journal of medicine. 2009;360(13):1298–309. 10.1056/NEJMoa0802556 19321868 PMC2676895

[10] Wawer MJ, Tobian AA, Kigozi G, Kong X, Gravitt PE, Serwadda D et al Effect of circumcision of HIV-negative men on transmission of human papillomavirus to HIV-negative women: a randomised trial in Rakai, Uganda. Lancet. 2011;377(9761):209–18. 10.1016/S0140-6736(10)61967-8 21216000 PMC3119044

[11] WHO, UNAIDS. Manual for male circumcision under local anaesthesia. WHO/UNAIDS/JHPIEGO, Version 3.1. 2009. Geneva, Switzerland, Department of Reproductive Health and Research;2009 December.

[12] UNAIDS. 2016–2021 Strategy: On the Fast-Track to end AIDS. Geneva, Switzerland: UNAIDS2016.

[13] United Nations. Transforming our World: the 2030 Agenda for Sustainable Development. New York, USA: United Nations, Department of Economic and Social Affairs/ Division for Sustainable Development;2015 Contract No.: A/RES/70/1.

[14] Geldsetzer P, Bloom DE, Humair S, Bärnighausen T. Benefits and Costs of the HIV/AIDS Targets for the Post-2015 Development Agenda: Copenhagen Consensus Center2015 March 11.

[15] WHO UNAIDS and UNICEF. Global HIV/AIDS Response: Epidemic update and health sector progress towards Universal Access. Progress report2011.

[16] Hallett TB, Singh K, Smith JA, White RG, Abu-Raddad LJ, Garnett GP. Understanding the impact of male circumcision interventions on the spread of HIV in southern Africa. PloS one. 2008;3(5):e2212 10.1371/journal.pone.0002212 18493593 PMC2387228

[17] Ministry of Health and ICF International. Uganda AIDS Indicator Survey 2011. Kampala, Uganda and Calverton Maryland, USA: MOH and ICF International2012.

[18] Kibira SP, Makumbi F, Daniel M, Atuyambe LM, Sandoy IF. Sexual Risk Behaviours and Willingness to Be Circumcised among Uncircumcised Adult Men in Uganda. PloS one. 2015;10(12):e0144843 10.1371/journal.pone.0144843 26658740 PMC4678174

[19] Uganda AIDS Commission. The HIV and AIDS Uganda progress report 2014. Kampala: UAC2015 June 15.

[20] World Health Organization. WHO Progress Brief: Voluntary Medical Male Circumcision for HIV Prevention in 14 Priority Countries in East and Southern Africa Geneva, Switzerland: WHO; 2015.

[21] Herman-Roloff A, Otieno N, Agot K, Ndinya-Achola J, Bailey RC. Acceptability of medical male circumcision among uncircumcised men in Kenya one year after the launch of the national male circumcision program. PloS one. 2011;6(5):e19814 10.1371/journal.pone.0019814 21603622 PMC3095626

[22] Jones D, Cook R, Arheart K, Redding CA, Zulu R, Castro J et al Acceptability, knowledge, beliefs, and partners as determinants of Zambian men's readiness to undergo medical male circumcision. AIDS and behavior. 2014;18(2):278–84. 10.1007/s10461-013-0530-0 23757123 PMC3815686

[23] Hilton H, Heidi vR, Lucia K, Ruanne B, Connie C. ‘If you are circumcised, you are the best’: understandings and perceptions of voluntary medical male circumcision among men from KwaZulu-Natal, South Africa. Culture, health & sexuality. 2015;17(7):920–31. 10.1080/13691058.2014.992045 PMC4470729 25567140

[24] Plotkin M, Castor D, Mziray H, Kuver J, Mpuya E, Luvanda PJ et al "Man, what took you so long?" Social and individual factors affecting adult attendance at voluntary medical male circumcision services in Tanzania. Glob Health Sci Pract. 2013;1(1):108–16. 10.9745/GHSP-D-12-00037 25276521 PMC4168557

[25] Cook R, Jones D, Redding CA, Zulu R, Chitalu N, Weiss SM. Female Partner Acceptance as a Predictor of Men's Readiness to Undergo Voluntary Medical Male Circumcision in Zambia: The Spear and Shield Project. AIDS and behavior. 2015. 10.1007/s10461-015-1079-x PMC4696907 25931242

[26] Mukama T, Ndejjo R, Musinguzi G, Musoke D. Perceptions about medical male circumcision and sexual behaviours of adults in rural Uganda: a cross sectional study. The Pan African medical journal. 2015;22:354 10.11604/pamj.2015.22.354.7125 26985272 PMC4779633

[27] Nevin PE, Pfeiffer J, Kibira SP, Lubinga SJ, Mukose A, Babigumira JB. Perceptions of HIV and Safe Male Circumcision in High HIV Prevalence Fishing Communities on Lake Victoria, Uganda. PloS one. 2015;10(12):e0145543 10.1371/journal.pone.0145543 26689212 PMC4686987

[28] Osaki H, Mshana G, Wambura M, Grund J, Neke N, Kuringe E et al "If You Are Not Circumcised, I Cannot Say Yes": The Role of Women in Promoting the Uptake of Voluntary Medical Male Circumcision in Tanzania. PloS one. 2015;10(9):e0139009 10.1371/journal.pone.0139009 26402231 PMC4581795

[29] Wirth KE, Semo BW, Ntsuape C, Ramabu NM, Otlhomile B, Plank RM et al Triggering the decision to undergo medical male circumcision: a qualitative study of adult men in Botswana. AIDS care. 2016:1–6. 10.1080/09540121.2015.1133797 26754167

[30] George G, Strauss M, Chirawu P, Rhodes B, Frohlich J, Montague C et al Barriers and facilitators to the uptake of voluntary medical male circumcision (VMMC) among adolescent boys in KwaZulu-Natal, South Africa. Afr J AIDS Res. 2014;13(2):179–87. 10.2989/16085906.2014.943253 25174635

[31] Uganda Bureau of Statistics. The National Population and Housing Census 2014- Main Report. Kampala, Uganda: UBOS2016.

[32] The Republic of Uganda. Ministry of Health. Health Facilities Inventory July 2012: Master Health Facilities Inventory. Kampala, Uganda: Ministry of Health, Department of Clinical Services;2012.

[33] Attride-Stirling J. Thematic networks: an analytic tool for qualitative research. Qualitative Research. 2001;1(3):385–405.

[34] Riess TH, Achieng MM, Bailey RC. Women's beliefs about male circumcision, HIV prevention, and sexual behaviors in Kisumu, Kenya. PloS one. 2014;9(5):e97748 10.1371/journal.pone.0097748 24844845 PMC4028254

[35] Ssekubugu R, Leontsini E, Wawer MJ, Serwadda D, Kigozi G, Kennedy CE et al Contextual barriers and motivators to adult male medical circumcision in Rakai, Uganda. Qualitative health research. 2013;23(6):795–804. 10.1177/1049732313482189 23515302

[36] Riess HT, Achieng MM, Otieno S, Ndinya-Achola JO, Bailey CR. “When I Was Circumcised I Was Taught Certain Things”: Risk Compensation and Protective Sexual Behavior among Circumcised Men in Kisumu, Kenya. PloS one. 2010;5(8):1–9. 10.1371/journal.pone.0012366 PMC2928269 20811622

[37] Shacham E, Godlonton S, Thornton RL. Perceptions of Male Circumcision among Married Couples in Rural Malawi. J Int Assoc Provid AIDS Care. 2014;13(5):443–9. 10.1177/2325957413508319 24162614

[38] Muhamadi L, Ibrahim M, Wabwire-Mangen F, Peterson S, Reynolds SJ. Perceived medical benefit, peer/partner influence and safety and cost to access the service: client motivators for voluntary seeking of medical male circumcision in Iganga district eastern Uganda, a qualitative study. The Pan African medical journal. 2013;15:117 10.11604/pamj.2013.15.117.2540 24255723 PMC3830467

[39] Zulu R, Jones D, Chitalu N, Cook R, Weiss S. Sexual Satisfaction, Performance, and Partner Response Following Voluntary Medical Male Circumcision in Zambia: The Spear and Shield Project. Glob Health Sci Pract. 2015;3(4):606–18. 10.9745/GHSP-D-15-00163 26681707 PMC4682585

[40] Fleming PJ, Barrington C, Pearce LD, Lerebours L, Donastorg Y, Brito MO. "I Feel Like More of a Man": A Mixed Methods Study of Masculinity, Sexual Performance, and Circumcision for HIV Prevention. J Sex Res. 2016:1–13. 10.1080/00224499.2015.1137539 PMC5011023 26942550

[41] Layer EH, Beckham SW, Mgeni L, Shembilu C, Momburi RB, Kennedy CE. "After my husband's circumcision, I know that I am safe from diseases": women's attitudes and risk perceptions towards male circumcision in Iringa, Tanzania. PloS one. 2013;8(8):e74391 10.1371/journal.pone.0074391 24009771 PMC3756960

[42] Westercamp M, Agot KE, Ndinya-Achola J, Bailey RC. Circumcision preference among women and uncircumcised men prior to scale-up of male circumcision for HIV prevention in Kisumu, Kenya. AIDS care. 2012;24(2):157–66. 10.1080/09540121.2011.597944 21854351 PMC3682798

[43] Johns Hopkins Bloomberg School of Public Health Center for Communication Programs. The Health Communication Partnership Uganda: 2007–2012 Final Report. Kampala, Uganda2012 May.

[44] Rakai Health Sciences Program and Johns Hopkins Bloomberg School of Public Health. A Guide for Community Games and Competitions Under the Stylish Man Campaign. RHSP and JHSPH Center for Communication Programs; 2012.

[45] The Republic of Uganda. Ministry of Health. Understanding Safe Male Circumcision for HIV Prevention [brochure]. In: Ministry of Health, editor. Kampala, Uganda2012.

[46] The Republic of Uganda. Ministry of Health. Safe male circumcision for HIV prevention. National communication strategy. Kampala, Uganda: Ministry of Health2010.

[47] Toefy Y, Skinner D, Thomsen SC. "What do You Mean I've Got to Wait for Six Weeks?!" Understanding the Sexual Behaviour of Men and Their Female Partners after Voluntary Medical Male Circumcision in the Western Cape. PloS one. 2015;10(7):e0133156 10.1371/journal.pone.0133156 26176946 PMC4503729

[48] Herman-Roloff A, Bailey RC, Agot K. Factors associated with the early resumption of sexual activity following medical male circumcision in Nyanza province, Kenya. AIDS and behavior. 2012;16(5):1173–81. 10.1007/s10461-011-0073-1 22052231 PMC3677080

[49] Kalichman S, Eaton L, Pinkerton S. Circumcision for HIV prevention: failure to fully account for behavioral risk compensation. PLoS medicine. 2007;4(3):e138; author reply e46. 10.1371/journal.pmed.0040138 17388676 PMC1831748

[50] Kong X, Ssekasanvu J, Kigozi G, Lutalo T, Nalugoda F, Serwadda D et al Male circumcision coverage, knowledge, and attitudes after 4-years of program scale-up in Rakai, Uganda. AIDS and behavior. 2014;18(5):880–4. 10.1007/s10461-014-0740-0 24633740

[51] L'Engle K, Lanham M, Loolpapit M, Oguma I. Understanding partial protection and HIV risk and behavior following voluntary medical male circumcision rollout in Kenya. Health education research. 2014;29(1):122–30. 10.1093/her/cyt103 24293524 PMC3894669

[52] Friedland BA, Apicella L, Schenk KD, Sheehy M, Hewett PC. How informed are clients who consent? A mixed-method evaluation of comprehension among clients of male circumcision services in Zambia and Swaziland. AIDS and behavior. 2013;17(6):2269–82. 10.1007/s10461-013-0424-1 23392912

[53] Maughan-Brown B, Godlonton S, Thornton R, Venkataramani AS. What Do People Actually Learn from Public Health Campaigns? Incorrect Inferences About Male Circumcision and Female HIV Infection Risk Among Men and Women in Malawi. AIDS and behavior. 2015;19(7):1170–7. 10.1007/s10461-014-0882-0 25155700

