# Supplementary material for: Exploring drivers for safe male circumcision: Experiences with health education and understanding of partial HIV protection among newly circumcised men in Wakiso, Uganda
Source: PLoS One. 2017 Mar 31;12(3):e0175228. doi: 10.1371/journal.pone.0175228 (PMC5376336; doi:10.1371/journal.pone.0175228)
Supplement: S1 Text — (DOCX) [file pone.0175228.s001.docx]

# Relevant Excerpts from Interviews

***Source Transcript: P17***

**I: How did you decide that you wanted to come for circumcision?**

I grew up with circumcising neighbours in my home district; the bagisu. I learnt a lot from them and wanted to be circumcised too then. Secondly, I fear contracting HIV and they say that circumcision reduces the chances of getting infection. so why don’t I circumcise with this big benefit. I also talked to my “women” and we agreed with them. I have been looking for suitable place to go. The first time I went, I found very many people at the facility, and the second time in another place, the doctors came late. Even at Kira Health centre, the time I went, the doctors were not around. Then I heard this public announcement in the community, and I decided that “on Monday, I will be the first person in the line”. When I came I found no doctor early in the morning, but at about midday, the doctors came and I was circumcised. it was my own will.

**I: You have said, you discussed this with your partners, what was their role in this decision?**

They were very happy because I told them I was going to circumcise. I haven’t called them now because they are all upcountry. But I will let them know that I have now done it.

**I: Okay, so what are your expectations, now that you have circumcised and we know its benefit?**

I don’t think I will change. I am not expecting any change even though I know the reason I circumcised was to reduce the risk of HIV. I will not look for new partners. You know the protection is not 100%. So you cannot say that you are safe after circumcision, there is still that chance that you can contract HIV. I have already tested for HIV here and I had tested with both of them. So I have to keep my behaviour.

**I: How about your wives, what are their expectations?**

I don’t know what they expect. but they are trustworthy.

-----------------------------------------------------------------------------------------------------

***Source Transcript: P9***

**I: Thank you for accepting to participate in this study and giving us your precious time. We will begin by discussing what people perceive of male circumcision in your home community.**

Mostly, they fear circumcision because they think of the pain involved and the time it will take them to heal. Many of them are paid for work done, living hand mouth and therefore would not want to take any time off. They think about the days it takes to heal and give up. You know these are hard times economically, thus not many people can afford time off work.

**I: Okay, what influences the men that go for circumcision?**

I will not talk for other people. I really don’t know. But for me I heard on the radio several times that circumcision helps to prevent some of the STIs like the ulcers if you have them. I had some recurring genital herpes and therefore decided to come for circumcision. That was my biggest challenge. I thought maybe this problem will go. I also hear that it prevents gonorrhoea.

**I: How about some of the young people you associate with?**

For young men, some of them get circumcised because their partners’ influence. The women tell say that when you are circumcised, everything will go well with you and any partner you are with ((seemingly shy)). That it helps to boost sexual performance. They claim that you find it easier when you are circumcised.

**------------------------------------------------------------------------------------------------------**

***Source Transcript: P1***

**I; What else? I would like you to tell me everything that influences men to come.**

Ha! I don’t know how to say this. ((Seemingly shy)) But a circumcised man makes sex much more pleasant for the partner. I think you understand what I am trying to mean here ((smiles)). They are not like the uncircumcised ones. I also hear this as an advantage of being circumcised, but I have not verified this since I have not yet had the chance to have sex after circumcision to confirm this claim. May be it is true.

**I: Okay, may be you will verify and tell us the next time we meet if this is true. Now we have talked about others. Let us talk about you in particular. What exactly influenced you to decide that I should now go for SMC? What other different factors influenced you that may be unique to you?**

For me, the very first reason was to reduce the chances of getting infected with HIV. The second reason was to reduce the dirt that builds up under the foreskin and also to reduce on the chances of my partner getting infected with those cancers I have told you ((cervical cancer)).

**I: So protecting your partner against cervical cancer was a big issue?**

Yes it was, but in a way I am also going to be protecting myself in the process.

**I: Could your partner have played a role in the decision to come for SMC?**

Yes, we had talked about it for some time. She used to ask me “why don’t you go for circumcision?” and I would ask her “why do you always ask me to go for it?” then she told me “you go get circumcised and you will see the reasons”. Now I have called her in the village and she is very excited that I have gone for it. She has promised to give me money when I go back home to see her ((laughter))

**I: Will she give you money only?**

Hehe ((all laugh)). She will give me other things also. Not just money. You know what I am talking about ((meaning sexual intercourse)).

**I: But what benefits was she talking about when she said ‘go for circumcision, you will see the benefits’?**

They are the same benefits, which I also knew and used to hear. The ones I have told you. She used to say the same things sometimes. Another reason she emphasised this is because she is a Muslim woman. So she wanted me to be circumcised and become and “Muslim” [okunsiramula] as well.

**I: In case your partner had not insisted on this, and since you had feared as well, would you have done it anyway without her influence?**

Yes I think I would. You know I had actually told her that I will never do it. So I came without telling her that I was finally going for it. I have just informed her about it after it was done. Then I can say I made the decision on my own.

**-------------------------------------------------------------------------------------------------**

***Source Transcript: P10***

**I: How about other people, what do they think?**

They fear to be circumcised. There is one I told yesterday that I have been circumcised and he said “you are more than a man. How do you go for circumcision? You need to have prepared a lot of money to help you during the healing process. How are you going to go through this?” he then said I should wait until the evening hours and I will feel the extreme pain. He really made me worried. But I have gone through the night. There is also another neighbour who is a Musoga. He is my tenant. I also told him I was circumcised and he was concerned. They all fear. He said he hates this programme.

The truth is that this is not painful, although I was scared as a human being. The only pain I felt was the anaesthesia injection. And the health workers were so helpful. I wish other health facility departments were as good as the circumcision department. They have a lot of care that you cannot find in other departments. They were young people but very caring.

There are still very many uncircumcised men, but convincing them to accept is the biggest challenge. I think most men fear the perceived pain involved.

**I: you have mentioned what influenced you, how about other men that you know. What influences them to come for circumcision?**

The other people have different reasons. Although the women may not say it out directly, but they all want circumcised men. They influence many men. For me, I did not tell any of my wives [has 3 wives] that I was going for circumcision, but when I told them that I had gone for it, they were all very excited yesterday. They praised me for taking the bold decision to go for it. Once said “eh, we have survived Salongo”. Imagine all along they wanted me to be circumcised but were not telling me directly. I think they want a clean man.

**I: Had any of your wives showed you any signs that they wanted you to be circumcised? or said it indirectly?**

Yes, they had. One of my wives, the Nalongo, told me one time that if I ever knew of cervical cancer screening programmes at the health facility, I should let her know. She said they had told them that an uncircumcised man has high chances of infecting their partners with a cervical cancer causing virus. Then she jokingly asked me “when will you ever gather courage and go for circumcision since you were able to take your sons for it?” So they wanted it but they would not come out directly to tell me.

May be something else I have not told you, they told me they only circumcise men who have no STI. That if I had an STI I had to be treated first. And they had to test me for HIV first.

**I: What are you expectations, now that you have been circumcised?**

In think circumcision should be compulsory for all sexually active men. If it has been proven that it is protective, then it should really be compulsory. In the future may be our children and other young men will be protected from all these infections that we have in the population now.

**----------------------------------------------------------------------------------------------------------------**

***Source Transcript: P8***

**I: what are the main influences of the men that go for circumcision?**

What I heard from my elder brother, he was infected with HIV and when he went to the health facility, he was told that it would be good for him to get circumcised to prevent further reinfection. That is what he told me. It is many years ago, about 10. So some people circumcise because of diseases.

I know that some young men who still look for sexual partners are influenced by the women who want them to be circumcised. Sometimes the girls put conditions of circumcision to allow any sexual relations with you. In such cases, the young man will run for circumcision. Even for older men, it is mainly because of the influence of women. The truth is that women influence a lot of men to get circumcised.

**I: Why do women influence them?**

I will give you several reasons. But I think mainly because it is fashionable to be circumcised nowadays for most women; it is the trend that many women want men to take. I would say it is like a woman telling you “please wear shorter socks, they are trendy, or you should not wear hemmed trousers.” It’s the way to go now. The reason I say this is because in the old days, women used to be in relationships and marriages with uncircumcised men comfortably, but now they want you to be circumcised in this era. It is very common. Many women tell men to get circumcised. But many of them I think have no main reason. It is just fashionable.

**I: Could there be some reason behind this “fashionable” trend?**

Women may say that it boosts the sexual performance of the man. But I don’t agree so much with this reason. We have always been uncircumcised and from time eternity, most men have not been circumcised. Why would someone raise this sexual performance reason now? Why now? So I don’t think it’s a valid reason, and I don’t support it.

**I: Okay, you also said, there are other reasons other than fashion.**

I have ever had a Muslim girlfriend before and she was insisting that I should get circumcised, but she had no concrete reason really. I think some could just want you to be circumcised because they are Muslim women.

The other reason they emphasise is cleanliness. This is a valid reason that women also front. I think I can support this one. When they were circumcising me today, they told me about this as well. When they showed us different penises in the demonstration pictures, I saw varied types and you could see that some of them have longer foreskins that harbour dirt. Of course you know it too. When a man is erect, the whole foreskin retracts back and all the dirt goes into the woman I think. May be that is the reason they do not like it. When you remove the foreskin, then all this dirt will not collect underneath. This is a very important influence that leads women to encourage men to circumcise.

**I: Okay, now let us come to your own decision. How did you decide that you want to be circumcised?**

I should be frank. I should I had the influence of my wife. Like I told you it is fashionable, and it was not appealing to her. I have spent 14 years with my wife. But you also notice your weakness and understand what she doesn’t like. You notice that she doesn’t want to look at your penis. For me to decide that I should go for it, I noticed that she is getting influence from other women about circumcision. She would bring up the topic of circumcision indirectly, wondering why men do not go for circumcision. And as a man who has spent a long-time with her, I noticed she was telling me indirectly what she wanted. So I gathered courage and decided to come and have it.

But also, because people used to claim that it enhances sexual performance, I decided to also test this notion and see if it is valid ((laughs)). The other reason I finally went, was because they told us that it is not a painful procedure. The AMREF team used to drive through our community with a loud speaker mobilising men to come for circumcision and telling us the procedure was not painful at all. This also helped me in making up my mind. But the message of reducing HIV infection risk by 60% was not appealing to me and I did not buy this reason. They also told us it prevents other diseases like penile cancer when I came here before circumcising us, but all these reasons did not influence me. I didn’t know all these benefits and I cannot say that they influenced me.

**I: tell me more about the 60% risk reduction you have mentioned and how you know it, what did they tell you about it?**

Sometime back, I read a piece in the newspaper, I don’t know if it was Bukedde or New vision [local dailies]. they had written about sexual performance and circumcision. That the head of the penis is very sensitive and when the foreskin retracts, it makes it much more sensitive and you cannot last long during intercourse. Of course any man would like to enhance their sexual performance. They said that if you get circumcised, that skin will be hardened and it will be less sensitive during intercourse. Now when they explained the 60% risk reduction here at the facility, I thought it is not necessarily related to the hardened foreskin. They say for HIV to be transmitted, it goes through the sexual fluids, and all penises have a urethral opening. This should be the easier passage for the virus. So I don’t believe that circumcision will reduce the chances of the virus entering through the opening on the penis tip because it still remains as open as that of an uncircumcised man. It is very hard to convince me that the 60% works. Even if the head of the penis is hardened, it is not that hard that nothing can enter through it. However, I did not challenge the doctor today because you know you cannot challenge health workers when they explain. And, no one can confirm that 60%, how do you confirm that the percentage is 60%? So I did not listen to this 60%. I am not convinced.

**-----------------------------------------------------------**

***Source transcript: P13***

**I; Okay, thank you. So now let us talk about the use of condoms among men in your community. How is the typical use of condoms?**

I think many people use condoms, those that are not married mostly.

**How about you?**

I use condoms personally. I use them with some women, not all. Sometimes you look at a girl and you are suspicious or have been hearing that she is having multiple partners. Then sometimes I do not use them especially if it is girl that I know well and have been tracking her sexual behaviour in the community or we have grown up together.

**Now that you are circumcised, how will your use of condoms be?**

I will keep using them as I have been. There are very many STIs, I may avoid HIV and then get gonorrhoea or any other STI. So I will not change my behaviour just because I am circumcised.

**Okay. Now as we come to an end, people have several beliefs about sex. Have you heard of any beliefs around sex and circumcision?**

I heard from my friend. He told me that sexual pleasure will be different once you are circumcised. That is what I know. He said that ‘your partner will be more satisfied and even if she cheated on you with an uncircumcised man, she will still come back to you because you are certainly better

-------------------------------------

***Source transcript: P6***

**I: what influences men to come for circumcision?**

I think most men are now circumcised. And we know that it reduces HIV risk. So when I think about, I think I should be circumcised. This has been the reason that has been pushing me too.

**I: okay, hmmm.**

Other men have been influenced by their wives or partners. They influence their decision. They keep telling them “go for circumcision”. But the main reason is like I have indicated, the fact that circumcision reduces the risk of HIV infection. I think that is the main reason that influences men to come for circumcision. They say that if you are not circumcised, you are liable to several infections. So that is the reason why I decided to come for it.

**I: Okay, lets us come to your decision that you are talking about now. Who influenced you?**

For me, it has been primarily my wife. She has been telling me all along to come for circumcision. But I also recognised it would be beneficial to me and I made the final decision to come.

**I: why did your wife want circumcision?**

The main reason for her was influencing me was hygienic. They say that an uncircumcised man is less hygienic and because of this, she kept insisting that I go as well and we discussed about it.

**I: how long has this discussion taken?**

It has taken about 3 months now. But today I decided to give her a surprise because she has gone to the village (her parents’ home). I want her to come back when “everything is new”. She is not expecting this at all because I have not done it for all the months we discussed. It will be a complete surprise. I have not even told her yet. And, I don’t want to tell her yet. I want her to find when all is well as she has always wanted.

**I: so would I be right to say she has played a key role?**

Yes, you are right. Although I had the idea but the other men I was associating with at work did not want it like I have told you. You know they say peer pressure can negatively influence your behaviour. So if it wasn’t for her, it would not have happened.

**I: You have indicated some of the benefits, but much as she influenced you, what was your primary reason for accepting?**

For me, when I look at circumcised man [penis], it looks really nice. That is the main reason I have decided to come so that if a woman sees me, they see a clean nice looking [penis].

**I: why did you decide to come when she is away?**

((laughs)) I have decided to wait until she is in the village away from me because now it will be easier for me to heal without any temptations. I will not be thinking about her when she is away. I have heard from men who have been circumcised, and they say when you think about a woman during the healing period, you get a lot of pain. And, this will delay your healing time. So I had to take this chance too.

**I: so what are your expectations in your life, now that you are circumcised?**

The changes that I expect::::: .((long silence)). The changes would be::: if I had higher chances of getting HIV infection, now that risk has been reduced. That is a change I think.

The other change is the way my wife has been seeing me in the past. My penis was not appealing to her, but now I think she will be excited and pleased to see me in the new state because I will be cleaner.

**------------------------------------------------------**

***Source transcript: P1***

**I: What else? I would like you to tell me everything that influences men to come.**

Ha! I don’t know how to say this. ((Seemingly shy)) But a circumcised man makes sex much more pleasant for the partner. I think you understand what I am trying to mean here ((smiles)). They are not like the uncircumcised ones. I also hear this as an advantage of being circumcised, but I have not verified this since I have not yet had the chance to have sex after circumcision to confirm this claim. May be it is true.

**I; Okay, may be you will verify and tell us the next time we meet if this is true. Now we have talked about others. Let us talk about you in particular. What exactly influenced you to decide that I should now go for SMC? What other different factors influenced you that may be unique to you?**

For me, the very first reason was to reduce the chances of getting infected with HIV. The second reason was to reduce the dirt that builds up under the foreskin and also to reduce on the chances of my partner getting infected with those cancers I have told you ((cervical cancer)).

**So protecting your partner against cervical cancer was a big issue?**

Yes it was, but in a way I am also going to be protecting myself in the process.

**--------------------------------------------------------------------------**

***Source transcript: P22***

**I: How about you, what influenced you?**

People have varied reasons for circumcising. I have been clean even before, and have had several sexual partners. I already have children from those relationships and I had no concern for circumcision then. But in the present time, if you have a woman that you love genuinely and have lived together for a long time, a man who is circumcised would certainly be more appealing even in a marriage. Better hygiene is one of the main reasons thus that I have decided to come for circumcision.

The other reason is my current wife. You know, you can have children with women but it doesn’t mean you love them so much. You may not mind that much even when they decide to leave you. You would not listen to everything they say. That is what happened may be in the past. But when you love your wife so much, you will agree to whatever she says. We agreed with my partner because she loved circumcision and I wanted to make her happy by circumcising since it also helps me. I have thought about it as well for some time. But I should say, I wasn’t forced by her and it is my own decision finally and I came alone, although I didn’t want to resist her idea because she wouldn’t be happy with me yet I love her.

**Apart from your partner, is there anything else or another person that influenced you?**

May be the other challenge is that I live in a Muslim community and a uncircumcised man wouldn’t fit in easily. I should say this is one of the minor reasons in addition to the other two. A circumcised man is more appealing. These days, since the government is sensitising the public about circumcision on TV and in our communities, people are able to see the dangers of not being circumcised. you know when you start to educate people about something, they will notice the disadvantages of what they have been doing or not doing. We were not concerned that much about uncircumcised men before but now many women talk negatively about uncircumcised men, which makes you hate yourself if you are one of them. Their [women] eyes have been opened by all this sensitisation. Sometimes women have this as a condition for having relationships with you. But also if you compare the appearance of two men if both states, a circumcised man has an advantage. The foreskin is not attractive at all given all the “dirt” associated with it.

**Is it right if I say that appearance was one of the influences from what you have said?**

Yes. I have been personally challenged about this even before my wife influenced me. If you do not bathe in the morning, and wait until the next evening, you will be surprised how dirty under your foreskin will be. In today’s slung language one would say “naawe obeera weetya” [you fear yourself]. You have to shower a minimum of twice a day, yet circumcised men can even shower once a day if they want without much trouble. I was tired of this.

**--------------------------------------------------------------------------------------**

***Source transcript: P8***

I will give you several reasons. But I think mainly because it is fashionable to be circumcised nowadays for most women; it is the trend that many women want men to take. I would say it is like a woman telling you “please wear shorter socks, they are trendy, or you should not wear hemmed trousers.” It’s the way to go now. The reason I say this is because in the old days, women used to be in relationships and marriages with uncircumcised men comfortably, but now they want you to be circumcised in this era. It is very common. Many women tell men to get circumcised. But many of them I think have no main reason. It is just fashionable.

**I: Could there be some reason behind this “fashionable” trend?**

Women may say that it boosts the sexual performance of the man. But I don’t agree so much with this reason. We have always been uncircumcised and from time eternity, most men have not been circumcised. Why would someone raise this sexual performance reason now? Why now? So I don’t think it’s a valid reason, and I don’t support it.

**I: Okay, you also said, there are other reasons other than fashion.**

I have ever had a Muslim girlfriend before and she was insisting that I should get circumcised, but she had no concrete reason really. I think some could just want you to be circumcised because they are Muslim women.

The other reason they emphasise is cleanliness. This is a valid reason that women also front. I think I can support this one. When they were circumcising me today, they told me about this as well. When they showed us different penises in the demonstration pictures, I saw varied types and you could see that some of them have longer foreskins that harbour dirt. Of course you know it too. When a man is erect, the whole foreskin retracts back and all the dirt goes into the woman I think. May be that is the reason they do not like it. When you remove the foreskin, then all this dirt will not collect underneath. This is a very important influence that leads women to encourage men to circumcise.

**I: Okay, now let us come to your own decision. How did you decide that you want to be circumcised?**

I should be frank. I should I had the influence of my wife. Like I told you it is fashionable, and it was not appealing to her. I have spent 14 years with my wife. But you also notice your weakness and understand what she doesn’t like. You notice that she doesn’t want to look at your penis. For me to decide that I should go for it, I noticed that she is getting influence from other women about circumcision. She would bring up the topic of circumcision indirectly, wondering why men do not go for circumcision. And as a man who has spent a long-time with her, I noticed she was telling me indirectly what she wanted. So I gathered courage and decided to come and have it.

But also, because people used to claim that it enhances sexual performance, I decided to also test this notion and see if it is valid ((laughs)). The other reason I finally went, was because they told us that it is not a painful procedure. The AMREF team used to drive through our community with a loud speaker mobilising men to come for circumcision and telling us the procedure was not painful at all. This also helped me in making up my mind. But the message of reducing HIV infection risk by 60% was not appealing to me and I did not buy this reason. They also told us it prevents other diseases like penile cancer when I came here before circumcising us, but all these reasons did not influence me. I didn’t know all these benefits and I cannot say that they influenced me.

**---------------------------------------------------------------------------------**

***Source Transcript: P3***

**I: Were you counseled when you went for circumcision?**

Yes, I was counseled.

**I: How was it? Tell me more about it.**

Before we were circumcised, we were first counseled, we were educated about HIV and what circumcision involves and we noticed that it was not a challenge. But when I went for HIV testing, we were asked, “what if you are found to be HIV positive, what will you do?” I thought to myself. If I am positive, I may just intentionally walk into the road to be hit by a car because this is a disease that does not have a cure. But I was counseled and after blood was drawn, tested and I was found to be HIV negative. But I had hope that I was negative. Although, you always have fear if you have had sex before.

**I: Did they counsel you for circumcision and HIV specifically?**

No.

**I: So you were counseled for only HIV and after that you just went for circumcision?**

Yes.

**I: What did they tell you before circumcision?**

They told us circumcision prevents STIs and HIV. They also told us that after circumcision, you are not supposed to have a full body shower for a whole week. You can only bathe partially, wipe your body and the groin area near the wound. Then after a week, if you want to shower, you get clean clear polythene and you wrap it around the private parts and bathe. After that you wipe yourself dry. You are not supposed to travel long distances for the first three days. You are not supposed to do very hard manual work. And other things I have forgotten.

**--------------------------------------------------------------------------------------------------**

***Source transcript: P9***

**I: How did you know about all these advantages of circumcision, especially risk reduction for STIs and HIV?**

I learnt from different sources but I also use my personal judgement and common sense. I have to educate myself as well. This policy of circumcising people has been here for many years, but the people who were circumcised at the beginning may have got some issues. I think there was need for more research and now things have improved a lot and I can trust the health workers doing the job than it was before. I think some men were having sex before the healing period in the beginning. But now I know it is supposed to be 6 weeks, and I want to spend my entire 6 weeks and then I will call the health workers to explain what I have gone through in case I have any problem after reducing sex.

**Did you have this information on risk reduction before coming here today?**

Yes, Yes. I have heard from several health workers who were circumcising in health facilities and in outreaches. They used to convey these messages during the mobilisation campaigns.

**What else do you remember as key messages from the circumcision counselling?**

The main message was caution not to resume sexual intercourse before the healing period of 6 weeks that they gave us. I think no married man will come out to complain that they have complications, because they were very clear on this message to all of us. Others were young children below boys. But anyone who had a girlfriend was cautioned on this.

The other message was hygiene throughout the healing period and after. The health worker who was at the reception of the tent cautioned us about hygiene, citing examples of old men he has come across who were really dirty.

**-------------------------------------------------------------------------------------------------**

***Source transcript: P8***

**I: before you came for circumcision, had you heard about this HIV risk reduction benefit of circumcision?**

Yes, I had heard about this before in the community. They used to drive through the community with loud speakers talking about this risk reduction benefit of male circumcision. I had also heard about this on radio. I listen to 93… Kingdom FM [a local Christian station] of pastor Kiganda, there was someone talking about it. That was 2 years ago. But even during that radio programme, people called in and wondered how the 60% is arrived at. They all argued that people are being misled, that men are going to change their behaviours because they know they will have their 60% chances which they can take advantage of. They will think “I have my 60% chances; I can be saved even if I have unprotected sex with an infected woman”.

**Okay, any other place?**

No. it was only those two sources.

**I: did you get counselling before being circumcised today?**

Yes, the health workers really did their work. I am very grateful to them. They counselled me very well, they told me everything about circumcision and how they actually do the procedure. They also told me how to look after the wound. They were very many things they told me. When I arrived, I found my other clients who had already been counselled. The counsellor told me to come in and sit although they had finished counselling the others. He asked if I wanted to be circumcised today. I told him I had only come for information, and then return another day. I thought that is the process. But he told me they circumcised daily, and I could get circumcised today. So he told me about all the advantages of circumcision; the diseases that it prevents, like penis cancer and cancer of the cervix for women, syphilis and many other diseases. After taking me through this, he took me through the process of circumcising, that I will be injected with only once, but he lied here because I was injected three times on the penis ((lots of laughter)). I think he didn’t want to scare me to fear that I will have several injections on the penis. He showed me different ways of circumcising but I did not mind any that they wanted to use. They checked if my penis was normal and also tested me for HIV. He said it was the requirement to get tested for HIV and be counselled. After, we signed some papers to confirm that we agree to be circumcised, just like these consent forms that we have signed with you.

**------------------------------------------------------------------------------------**

***Source transcript: P20***

**I: Okay, let us talk about the role of circumcision in reducing HIV risk. You talked of some 60% before.**

Yes, they taught us about how circumcision reduces the risk of HIV infection. they indicated that it reduces by 60% the chances that you will be infected in case you have sex with an infected woman. It also reduces the chances of getting STDs.

**How do you understand this?**

When you have unprotected sex with a woman, there is always some form of friction especially if the woman is not well lubricated. You may be bruised in the process. So to some extent the circumcision reduces the risk of being bruised.

**Do you think other men understand this the same way?**

Certainly not all men understand this. But many of my friends do understand this and encourage other men to go.

For those who do not understand, it may be because of the way it is explained. If you just explain this in English, not everyone will understand it. The problem may therefore be with our “teachers” [counsellors].

**Now that you are circumcised, what do you expect especially in behaviour?**

Before they circumcision, they first test you for HIV and you receive your results. If you are HIV negative like me and then have the additional protection of male circumcision, then it gives you more courage to even behave better.

**Where did you get information regarding HIV risk reduction as a benefit of circumcision?**

I first heard about this when I was at the university. We used to have education talks about circumcision. That was at the time when they were encouraging people to go for circumcision and informing them of its benefit. I have also heard this on the radio and then my peers. i think on capital radio, there was a show called “capital doctor” where people would ask questions about health.

**Did you receive any counselling when you were circumcised?**

Not really, but they told us about how to behave after circumcision. They told us that circumcision alone will not prevent HIV completely or other STIs, but it reduces the risk by 60%. So they encouraged us to behave well. They also told us about how to behave during the healing process so that we have no complications.

**------------------------------------------------------------------------------------------------------**

***Source Transcript: P11***

**I: Okay. Let us now talk about circumcision and the relationship with HIV risk reduction. Tell me about how circumcision reduces HIV infection risk?**

They say that it reduces HIV risk. I heard that it reduces by 60%. This means though that the 40% chances are still existent. You know even in football if one team presses and wins possession for 60 minutes in a game, they may still lose the game when the other team only had 30 minutes of possession. That is how I relate this risk reduction in normal life. It means you can still get infected with HIV in case you do not use condoms if you think you will depend on the 60% chances alone. It means you still have to protect yourself once you know you are HIV negative. We all want life, especially if you are a young person and have no child in life yet.

**How do other people understand this risk reduction?**

I do not know really, but I think they understand it. Some may come for circumcision and have no partner and will still wait until they are ready for marriage. Others circumcise for hygiene or other reasons and may not mind about the HIV risk reduction. Like one of my colleagues has no partner and he is doing this because of hygiene [one of the three that he came with].

**Where did you hear about this risk reduction from?**

I had this from the radio, they advertise this. I also hear from the community when the drive through with loud speakers asking people to go for circumcision with free transport to and from. They also talk about risk reduction as they mobilise people.

**Anywhere else?**

No.

**Okay, now let us talk about counselling. Have you received any counselling when you came here?**

No, they did not tell us anything.

**Could you tell us what happened when you entered the facility up to now?**

We entered and told them we have come for circumcision. They told us to buy books, and then showed us the health worker responsible who wrote something in the books and we were sent to the HIV testing lab, we were tested and received results. We then came back to the circumcision theatre and that was it.

**Why do you think they did not explain anything to you?**

May be the counsellors are not around. I do not know really. You know what happens with our public health systems.

**----------------------------------------------------------------------------------------------------**

***Source Transcript: P11***

**I: Okay. Let us now talk about circumcision and the relationship with HIV risk reduction. Tell me about how circumcision reduces HIV infection risk?**

I heard it reduces [HIV risk] by 60%. This means that the 40% chances [to be infected] are still there. Even in a football game, if one team has 70% ball possession, they may still lose the game when the other team has only 30% of the ball. That is how I relate this risk reduction in normal life. It means you can still get infected in case you do not use condoms if you think you will depend on the 60% chances alone. It means you still have to protect yourself when you know you are HIV negative. We all want life. Especially when you are still a young person and have no child in life yet

**How do other people understand this risk reduction?**

I do not know really, but I think they understand it. Some may come for circumcision and have no partner and will still wait until they are ready for marriage. Others circumcise for hygiene or other reasons and may not mind about the HIV risk reduction. Like one of my colleagues has no partner and he is doing this because of hygiene [one of the three that he came with].

**Where did you hear about this risk reduction from?**

I had this from the radio, they advertise this. I also hear from the community when the drive through with loud speakers asking people to go for circumcision with free transport to and from. They also talk about risk reduction as they mobilise people.

**Anywhere else?**

No.

**-----------------------------------------------------------------------------------------------**

***Source Transcript: P1***

**I: What exactly do you know about this? How does it reduce your chances of getting HIV infection.**

You see, when you are circumcised, because this foreskin is now removed, the head of your penis becomes hardened. So the HIV virus cannot enter the skin that is hardened unlike the uncircumcised man. This means you have reduced your chances of the virus entering your skin.

**how about other ways that this removal helps?**

It reduces on the dirt that builds up under the foreskin. When you are hardened, you cannot have bruises on the head of the penis, you get rid of all these potential damages to the skin that you would get if you were not circumcised. It is so easy to get bruises on the skin when you engage in sexual intercourse with your partner if uncircumcised. But this is very hard for a circumcised man, because you cannot bleed. When you are not circumcised you bleed because you get bruised and this is a big risk for HIV infection.

**About what you have heard, have you had specifically about reducing risk by 60%?**

Yes I have heard about this

**So how do you understand it in your own way? What does it mean to you?**

for me in my own understanding, I see that all these other ‘minor’ diseases, the STDs, like syphilis and gonorrhoea, you actually get rid of all the risks for such. They cannot infect you at all with these ‘minor’ STDs.

**But the 60% chances are for HIV specifically? Haven’t you had about this on radio as well?**

Yes I hear about this on radio actually. *((Rain interrupted the interview and we moved to the house pouch)).*

**I hope we can talk freely here as well.**

Yes we can. They will not hear us. Actually the people I stay with do not know that I have gone for circumcision.

**-------------------------------------------------------------------------------------**

***Source Transcript: P5***

**I: You have talked about your wife as well, what was her role in this?**

My wife was also very interested in circumcision. We had discussed this with her as well, she had this idea even if she is not Muslim. She told me it is true that a man who is circumcised is really clean and is very good in sexual intercourse. We also want to circumcise the children. I have asked if they circumcise children too and they say it is possible.

**I: So did she influence you?**

Not really, You know women in most cases fear to directly “force” their ideas to their partners. But in my mind I had it. We have not spent a long time together, we have only one child together. When she became pregnant, we started living together. But she has been wanting it as well when we talked about it. So she is happy to know that I have been circumcised.

**I: Now, if we look ahead. What are your expectations now that you are circumcised.**

I haven’t thought about any changes because I am now still “sick” (wound). But I think when I am healed; it will now help me reduce chances of getting infected because I can just wipe my penis after intercourse to prevent STIs. I will be different. The other thing I expect, I have hope that finally my dream of preventing STIs that I feared, most especially gonorrhoea, is now realized. I am taking this [circumcision] as being vaccinated against those STIs, as I always wanted.

**I: You have said, circumcision changes the way a man performs sexually, how will this change your behavior.**

I should say I will keep the same but my intent in this case for is may be to make my wife happier and more satisfied sexually than I was doing before. It doesn’t mean I will be like my grandfather and get other wives because I am not a Muslim.
